# Supplementary material for: Rap1 prevents colitogenic Th17 cell expansion and facilitates Treg cell differentiation and distal TCR signaling
Source: Commun Biol. 2022 Mar 4;5:206. doi: 10.1038/s42003-022-03129-x (PMC8897436; doi:10.1038/s42003-022-03129-x)
Supplement: Supplementary file 2 — Supplementary information [file 42003_2022_3129_MOESM2_ESM.pdf]

Supplementary Fig.1

a

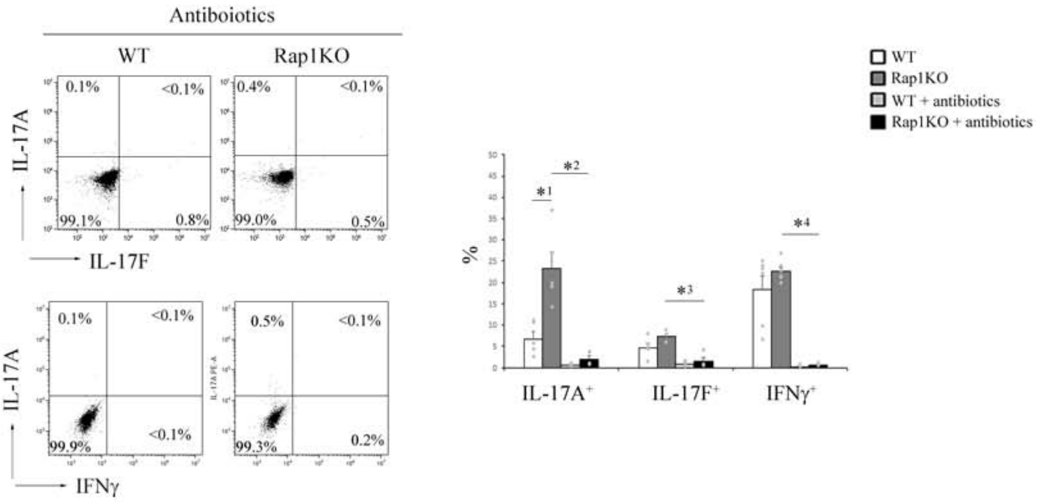

b

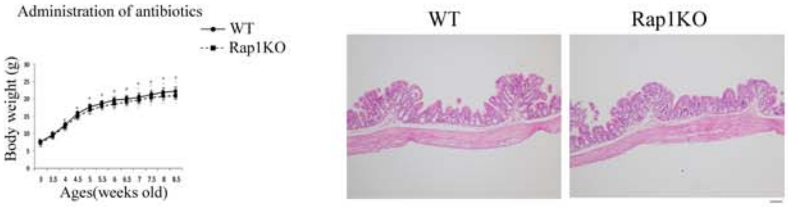

c

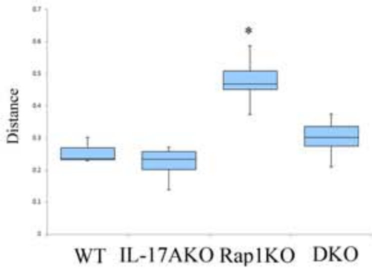

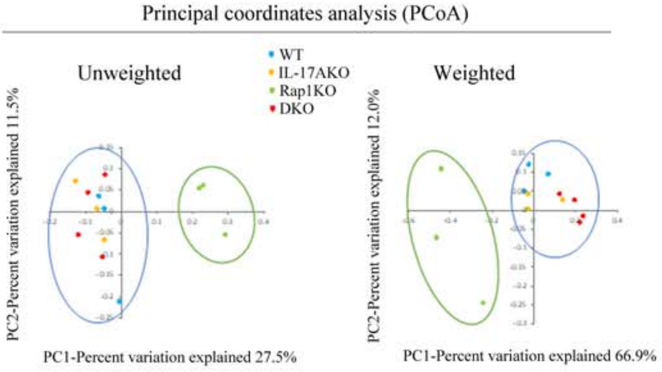

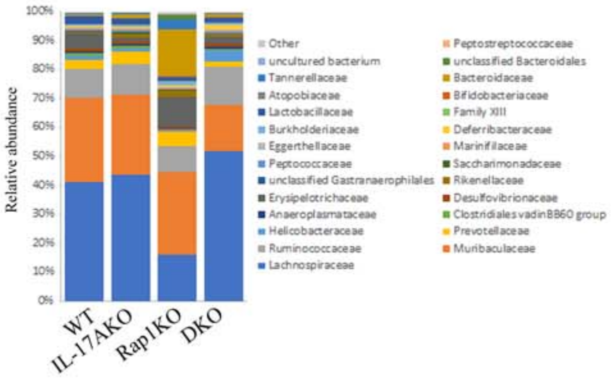

Decreased in Rap1KO mice

family	relative frequency average(%) ± SE				p-value
	WT	Rap1KO	IL-17KO	DKO	
Lachnospiraceae	41.12 ± 6.48	15.96 ± 9.16	43.63 ± 6.31	51.98 ± 3.30	0.0002
Clostridiales vadinBB60 group	1.02 ± 0.21	0.02 ± 0.02	0.78 ± 0.21	0.3 ± 0.16	0.0007

Increased in Rap1KO mice

family	relative frequency average(%) ± SE				p-value
	WT	Rap1KO	IL-17KO	DKO	
Rikenellaceae	0.46 ± 0.20	2.16 ± 1.31	1.28 ± 0.40	0.91 ± 0.23	0.0009
Family XIII	0.10 ± 0.07	0.29 ± 0.08	0.14 ± 0.06	0.13 ± 0.04	0.0042
Bacteroidaceae	0.64 ± 0.56	15.94 ± 6.40	1.38 ± 1.07	1.06 ± 0.69	0.0183
unclassified Bacteroidales	0.05 ± 0.09	1.64 ± 0.65	0.02 ± 0.02	0.02 ± 0.02	0.0208
Christensenellaceae	0.03 ± 0.04	0.13 ± 0.05	0.01 ± 0.01	0.02 ± 0.02	0.0499

Supplemental Fig. 1 Rap1KO mice developed the colitis in a microbiota-dependent manner.

(**a**) (Left) Representative IL-17A and IL-17F profiles, and IL-17A and IFNγ profiles of $CD4^+$ cells from the LILP of WT and Rap1KO mice that were administered antibiotics at 8 weeks of age ($n = 3$-6). (Right) Graphs represent the mean ± S.E.M. ratios of IL-17A-expressing, IL-17F-expressing, and IFNγ-expressing cells to $CD4^+$ cells in the LILP. $*^1P < 0.001$ compared with WT mice. $*^2P < 0.001$, $*^3P < 0.02$ and $*^4P < 0.001$ compared with Rap1 KO mice that were not administrated.

(**b**) (Left) The body weights of WT and Rap1KO mice that were administered antibiotics ($n = 3$) were measured every week. Data represent the mean ± S.E.M. (Right) Representative histology of intestinal inflammation. Paraffin-embedded LI sections from WT and Rap1KO mice that were administered antibiotics were stained with hematoxylin and eosin. Representative low (40×) magnification histological images of LI tissues are shown. Scale bars, 200 μm.

(**c**) Gut microbiome analysis of WT, Rap1 KO, IL-17A KO, and DKO mice. (Upper left) Comparison of gut microbiome profiles based on weighted UniFrac distances between WT, IL-17A KO, Rap1 KO and DKO mice. $*P < 0.001$ compared with WT mice. (Upper right) Unweighted and weighted UniFrac PCoA comparing the gut microbiome profiles of WT, Rap1 KO, IL-17A KO, and DKO mice.

(Lower left) Bar graph showing the relative abundance of abundant family (average abundance in all samples >0.1%) in WT, IL-17A KO, Rap1 KO and DKO mice ($n = 3$-12). (Lower right) Bacterial family of gut microbes which presence differed significantly between WT and Rap1KO mice.

Supplementary Fig.2

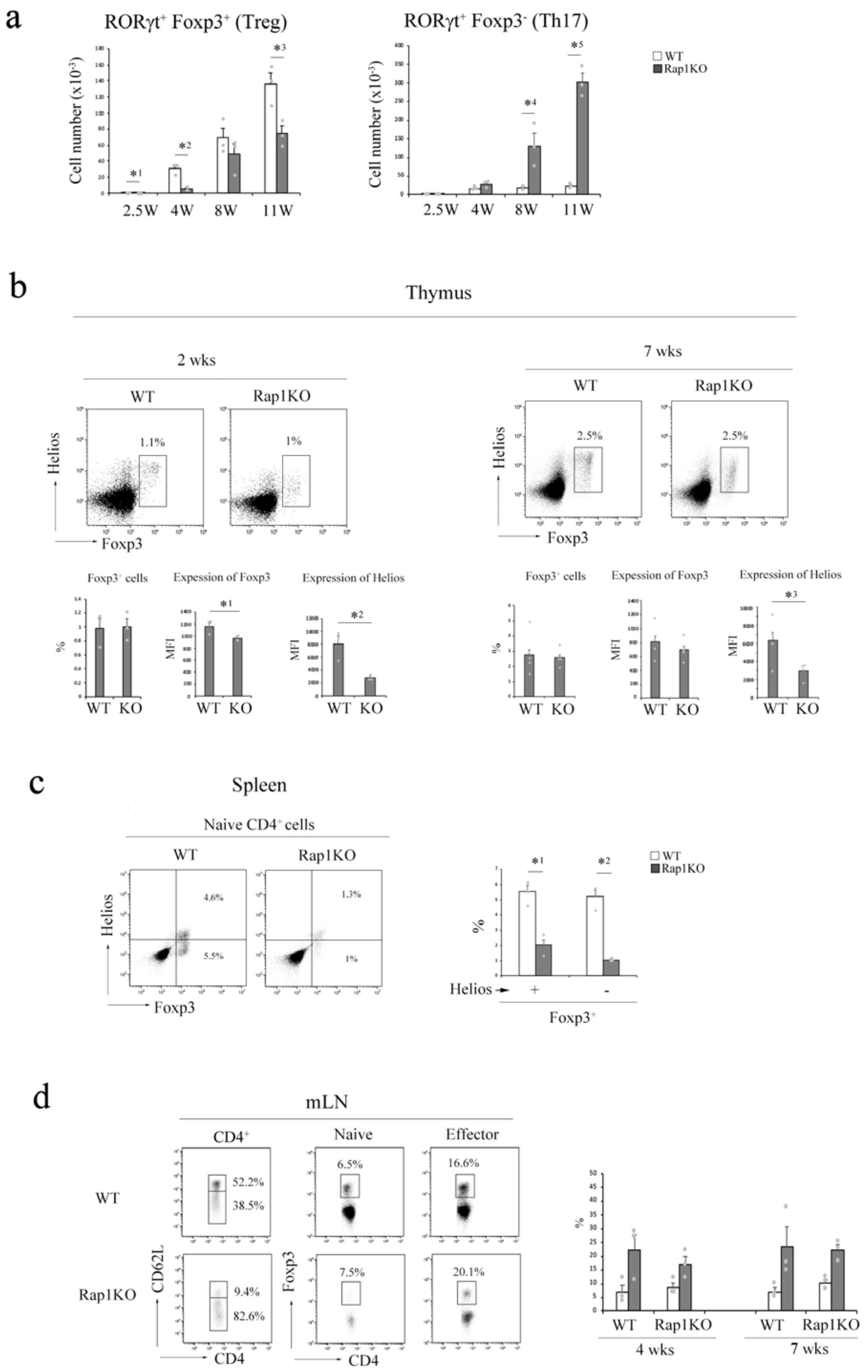

Supplemental Fig. 2 Rap1 deficiency impaired the generation of Treg cells.

(**a**) Numbers of RORγt^{+}Foxp3^{+} cells (left) and RORγt^{+}Foxp3^{-} cells (right) among CD4^{+} cells in the LILP of WT and Rap1KO mice at 2.5~11 weeks of age ($n = 3$). Data represent the mean ± S.E.M. $*^{1}P < 0.05$, $*^{2}P < 0.004$, $*^{3}P < 0.03$, $*^{4}P < 0.03$ and $*^{5}P < 0.001$ compared with the corresponding WT mice.

(**b**) (Upper) Representative flow cytometric profiles of Helios and Foxp3 in naïve CD4^{+} cells in the thymus of WT and Rap1KO mice at 2 and 7 weeks of age. (Lower) The percentages of Foxp3^{+} cells (left) and MFIs of Foxp3 (center) and Helios (right) in Helios^{+} Foxp3^{+} cells ($n = 3$-4). Graphs represent the mean ± S.E.M. $*^{1}P < 0.03$, $*^{2}P < 0.03$, and $*^{3}P < 0.05$ compared with WT mice.

(**c**) (Left) Representative flow cytometry profiles of Helios and Foxp3 on naïve CD4^{+} cells in the spleen of WT and Rap1KO mice at 4 weeks of age. (Right) The percentages of Helios^{+} Foxp3^{+} and Helios^{-} Foxp3^{+}cells to CD4^{+} cells ($n = 3$-4). Graphs represent the mean ± S.E.M. $*^{1}P < 0.002$ and $*^{2}P < 0.001$ compared with WT mice.

(**d**) (Left) Representative flow cytometry profiles of Foxp3 on naïve and effector CD4^{+} cells in the mLNs of WT and Rap1KO mice at 7 weeks of age. (Right) The percentages of Foxp3^{+} cells among CD4^{+} cells in the in the mLNs of WT and Rap1KO mice at 4 and 7 weeks old ($n = 3$). Graphs represent the mean ± S.E.M.

Supplementary Fig.3

a

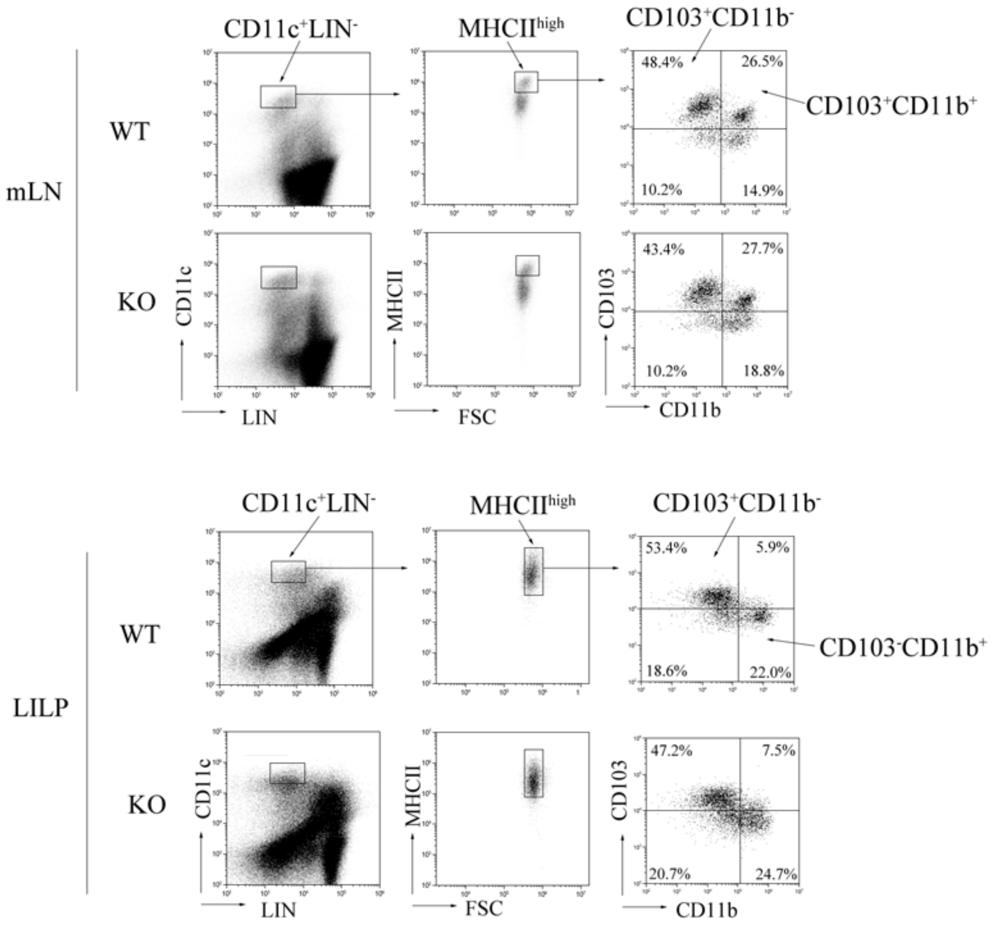

b

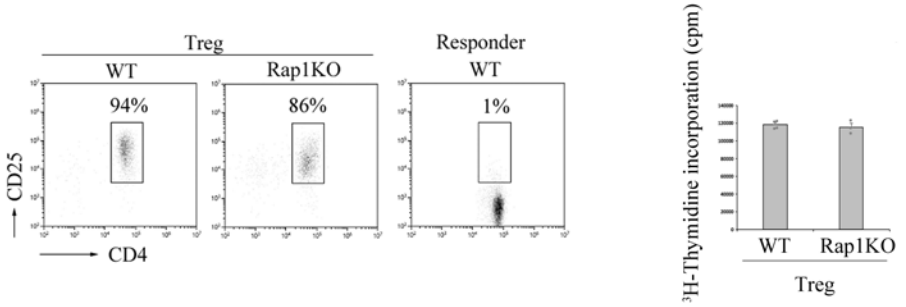

Supplemental Fig. 3 Flow cytometric analysis of DCs and Treg cells from WT and Rap1KO mice**.**

(**a**) Representative flow cytometry profiles of LIN (lineage; CD3, CD19, B220, NKp46, F4/80 and CD64) and CD11c (left), MHCII and FSC profiles of $CD11c^{+}LIN^{-}$ cells (center), $CD103^{+}$ and $CD11b^{+}$ profiles of $CD11c^{+}$ $MHCII^{high}$ cells (right), from the mLN (upper) and LILP (lower) of WT and Rap1KO mice.

(**b**) (Left) Representative CD25 and CD4 profiles of the purified WT responder cells, and Treg cells from spleen of WT and Rap1KO mice using $CD4^{+}CD25^{+}$ Treg cell isolation kit, mouse. (Right) [^{3}H]-Thymidine uptake by purified Treg cells from spleen of WT and Rap1KO mice. Treg cells were stimulated with 0.5 μg/ml of anti-CD3 in the presence of anti-CD28 for 48 h. [^{3}H]-Thymidine uptake was measured in triplicate. Data represent the mean ± S.E.M.

Supplementary Fig.4

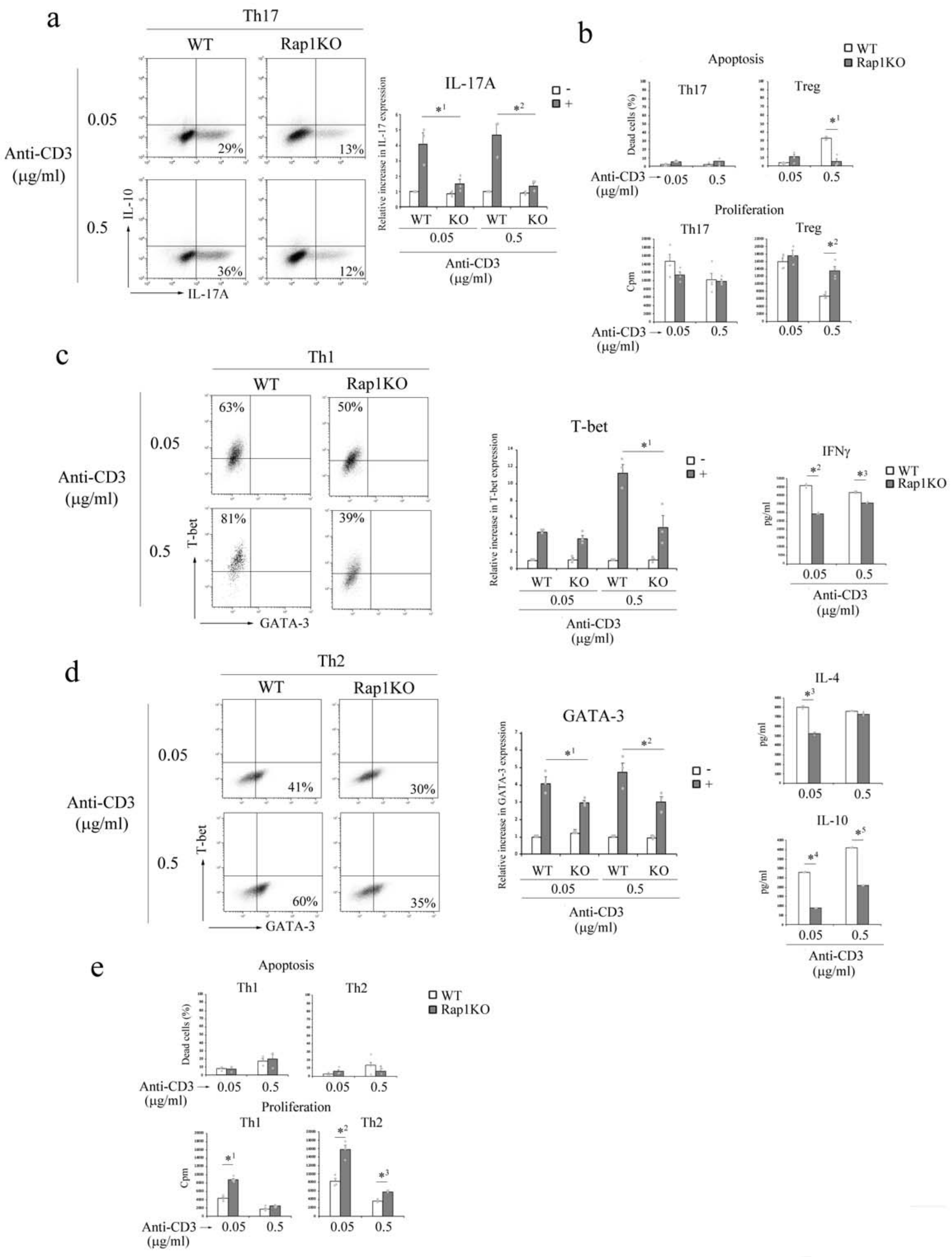

Supplemental Fig. 4 The differentiation of Rap1-deficient naïve $CD4^+$ cells was defective *in vitro*.

(**a**) (Left) Representative IL-10 and IL-17A profiles of naïve $CD4^+$ cells from WT and Rap1KO mice; the cells were cultured for 3 days under Th17-polarizing conditions in the presence of different concentrations (0.05 and 0.5 μg/ml) of anti-CD3 and 2.5 μg/ml of anti-CD28 and stimulated for 4 h with PMA plus ionomycin, subjected to flow cytometry in order to determine the prevalence of IL-17A$^+$ cells among $CD4^+$ cells. Data are representative of three independent experiments. (Right) The induction of IL-17A$^+$ cells in WT and Rap1-deficient naïve $CD4^+$ cells is presented as the fold increase in the normalized MFI of IL-17A$^+$ in the cells cultured under Th17-polarizing conditions (+) relative to that on WT naïve $CD4^+$ cells cultured with anti-CD3 and anti-CD28 (−) (adjust 1). Graphs represent the mean ± S.E.M ($n = 3$). $*^1P < 0.03$ and $*^2P < 0.02$ compared with the corresponding WT naïve $CD4^+$ cells.

(**b**) WT and Rap1-deficient naïve $CD4^+$ cells were stimulated with 0.5 or 0.05μg/ml of anti-CD3 in the presence of anti-CD28 under Th17 or Treg-polarizing condition. Apoptosis was measured by the incorporation of zombie dye (upper) ($n = 3$-4). Proliferation was measured by [^{3}H]-Thymidine uptake in triplicate or quadruplicate (lower). Data represent the mean ± S.E.M. $*^1P < 0.001$, $*^2P < 0.002$ compared with the corresponding WT naïve $CD4^+$ cells.

(**c**) (Left) Representative T-bet and GATA-3 profiles of naïve $CD4^+$ cells under Th1-polarizing conditions. Naïve $CD4^+$ cells from the spleen of WT and Rap1KO mice were cultured for 4 days under Th1-polarizing conditions in the presence of different concentrations (0.5 and 0.05 μg/ml) of anti-CD3 and 2.5 μg/ml of anti-CD28 and subjected to flow cytometry in order to determine the prevalence of T-bet$^+$ cells among $CD4^+$ cells. Data are representative of three independent experiments. (Center) The induction of T-bet in WT and Rap1-deficient naïve $CD4^+$ cells is presented as the fold increase in the normalized MFI of T-bet expressed on the cells cultured under Th1-polarizing conditions (+) relative to that expressed in WT naïve $CD4^+$ cells cultured with anti-CD3 and anti-CD28 (−) (adjust 1). Graphs represent the mean ± S.E.M ($n = 3$). $*^1P < 0.02$ compared with the corresponding WT naïve $CD4^+$ cells. (Right) WT and Rap1-deficeint cells which were cultured for 4 days under Th1-polarizing conditions were washed, and stimulated for 4 h with PMA plus ionomycin, and the amounts of IFNγ in the supernatants were measured using CBA (cytometaric Bead Array) kit. Graphs represent the mean ± S.E.M ($n = 3$). $*^2P < 0.001$, $*^3P < 0.001$ compared with the corresponding WT naïve $CD4^+$ cells.

(**d**) (Left) Representative T-bet and GATA-3 profiles of $CD4^+$ cells under Th2-polarizing conditions. Naïve $CD4^+$ cells from the spleen of WT and Rap1KO mice were cultured for 4 days under Th2-polarizing conditions in the presence of different concentrations of anti-CD3 and anti-CD28 and subjected to flow cytometry in order to determine the frequency of $GATA3^+$ cells among $CD4^+$ cells. Data are representative of three independent experiments. (Center) The induction of GATA3 in WT and Rap1-deficient $CD4^+$ cells is presented as the fold increase in the normalized MFI of GATA-3 expressed in the cells cultured under Th2-polarizing conditions (+) relative to that expressed on WT $CD4^+$ cells cultured with anti-CD3 and anti-CD28 (−) (adjust 1). Graphs represent the mean ± S.E.M (n = 3). $*^1P < 0.05$ and $*^2P < 0.05$ compared with the corresponding WT naïve $CD4^+$ cells. (Right) WT and Rap1-deficeint cells which were cultured for 4 days under Th2-polarizing conditions were washed, and stimulated for 4 h with PMA plus ionomycin, and the amounts of IL-4 and IL-10 in the supernatants were measured using CBA kit. Graphs represent the mean ± S.E.M (n = 3). $*^3P < 0.001$, $*^4P < 0.001$ and $*^5P < 0.001$, compared with the corresponding WT naïve $CD4^+$ cells

(**e**) WT and Rap1-deficient naïve $CD4^+$ cells were stimulated with 0.5 or 0.05μg/ml of anti-CD3 in the presence of anti-CD28 under Th1 or Th2-polarizing condition. Apoptosis was measured by the incorporation of zombie dye (upper). Proliferation was measured by $[^3H]$-Thymidine uptake in triplicate or quadruplicate (lower). Data represent the mean ± S.E.M. $*^1P < 0.001$, $*^2P < 0.001$ and $*^3P < 0.001$ compared with the corresponding WT naïve $CD4^+$ cells.

Supplementary Fig.5

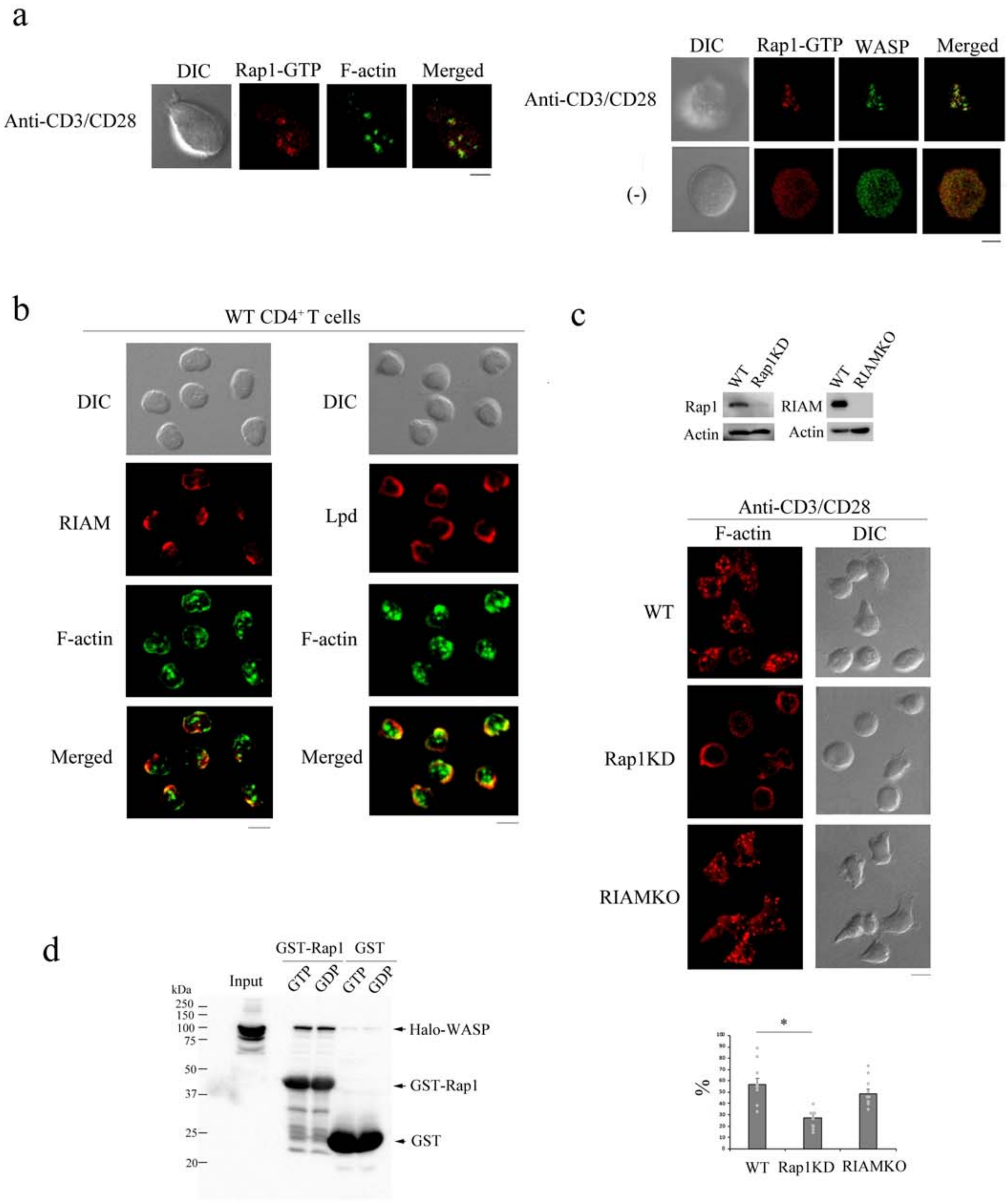

Supplemental Fig. 5 The regulation of actin foci by Rap1-signaling.

(**a**) (Left) Co-localization Rap1-GTP and actin foci. 3A9 T cells were transfected with Ral-GDS-RBD-mCherry as a reporter of Rap1GTP and Lifeact-GFP as a reporter of F-actin. They were stimulated on glass coated with anti-CD3 and anti-CD28 at 37°C for 5 min, then the digital images of contact areas of the cells were taken. (Right) Co-localization Rap1-GTP and WASP. 3A9 T cells were transfected with Ral-GDS-RBD-mCherry and WASP-GFP. They were stimulated on glass coated without or with anti-CD3 and anti-CD28 at 37°C for 5 min, then the digital images of contact areas of the cells were taken.

(**b**) Localization of RIAM and Lpd in $CD4^+$ T cells. WT naïve $CD4^+$ cells stimulated with anti-CD3 and anti-CD28 for 10 min were stained with Alexa488-phalloidin (green) and anti-RIAM or anti-Lpd (red). Representative images are shown. Scale bar, 5 μm.

(c) Actin polymerization in RIAM-deficient 3A9 T cells. (Top) The immunoblotting of WT and Rap1-knockdown (KD), or RIAM-knockout (KO) cells with anti-Rap1(left) or anti-RIAM (right). Actin is a loading control. (Middle) WT, Rap1-KD and RIAM-KO cells were incubated on glass coated with anti-CD3 and anti-CD28 for 10 min, fixed, stained for F-actin using Alexa488-phalloidin (red), and imaged using confocal microscopy. Representative images of two experiments are shown. Scale bar, 5 μm. (Bottom) Graphs represent the mean ± S.E.M. of the percentages of WT Rap1-KD and RIAM-KO cells showing F-actin clusters (actin foci) ($n = 30$). *$P < 0.001$ compared with WT 3A9 T cells.

(**d**) Association between Rap1 and WASP. Lysates from COS cells transfected with Halo-WASP were pulled down using GST fusions of Rap1 or GST during the loading of GTPγS or GDPγS and immunoblotted with anti-Halo or anti-GST.

Supplementary Fig.6

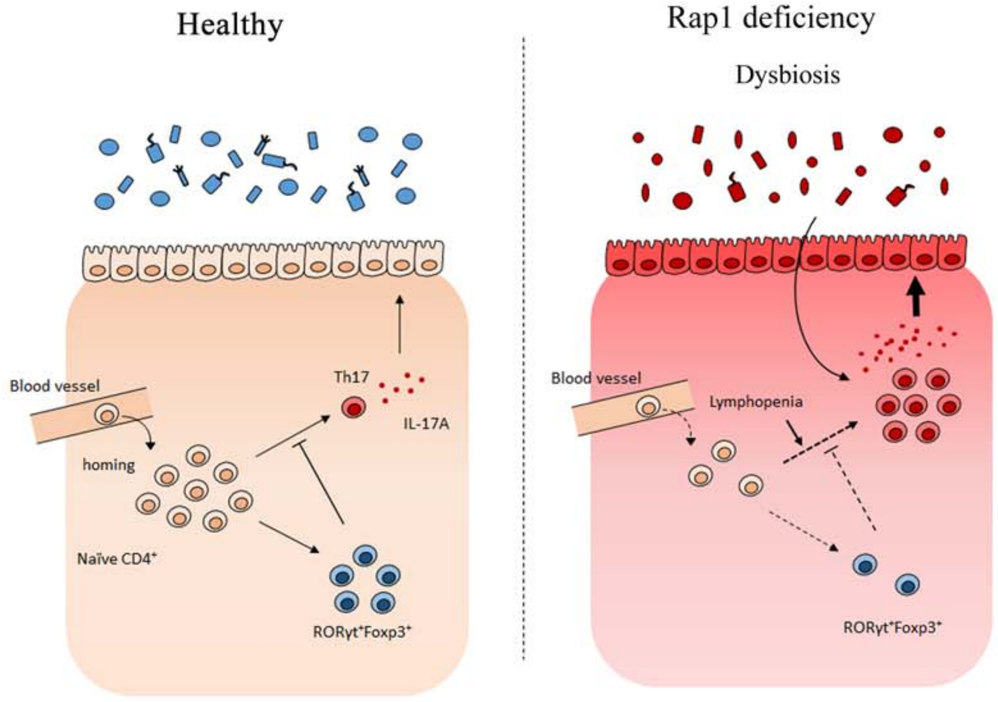

Supplemental Fig. 6 Model for the generation of colitogenic Th17 cells in the LILP of Rap1KO mice.
(Left) Naïve $CD4^+$ cells home into LILP of WT mice in integrin-dependent manner, and predominantly differentiate into $ROR\gamma t^+$ $Foxp3^+$ cells by 4 weeks of age in TCR-dependent manner in response to microbiota. $ROR\gamma t^+$ $Foxp3^+$ cells suppress the generation of microbe-reactive Th17 cells in the LILP of WT mice. The number of Th17 cells are appropriately maintained.
(Right) Rap1-deficiency impairs the homing of naïve $CD4^+$ cells, and reduces TCR-dependent induction of Foxp3 in naïve $CD4^+$ cells in the LILP. In the absence of sufficient suppression by $ROR\gamma t^+$ $Foxp3^+$ cells, lymphopenia accelerates the generation and expansion of Th17 cells and leads to the intestinal inflammation. Dysbiosis and impaired epithelial barrier functions might enhance the production of IL-17.

Supplementary Fig.7

Figure 6d

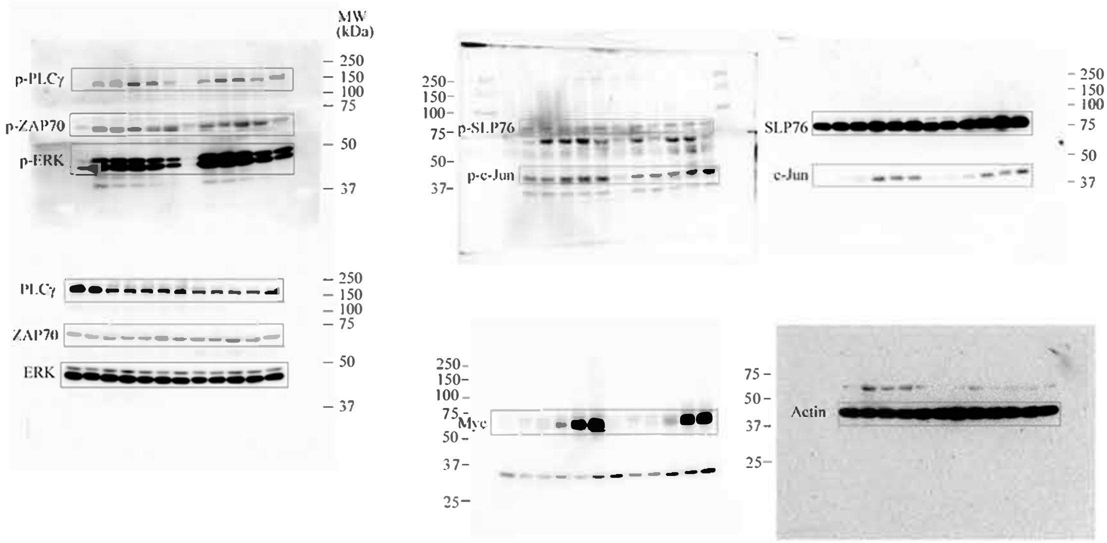

Figure 7b

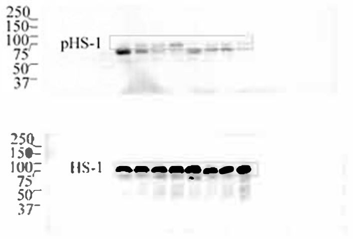

Supplementary Fig.5c

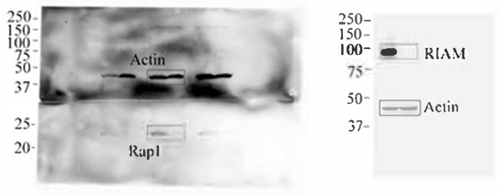

Supplementary Fig.7. Full blots for immunoblotting shown in Figures and Supplementary Figures.
