# Supplementary material for: Rap1 prevents colitogenic Th17 cell expansion and facilitates Treg cell differentiation and distal TCR signaling
Source: Commun Biol. 2022 Mar 4;5:206. doi: 10.1038/s42003-022-03129-x (PMC8897436; doi:10.1038/s42003-022-03129-x)
Supplement: Supplementary file 4 — Description of Additional Supplementary Files [file 42003_2022_3129_MOESM4_ESM.pdf]

Description of Additional Supplementary Files

File name: Supplementary Data 1
Description: Source data for graphs.
