# Supplementary material for: Rap1 prevents colitogenic Th17 cell expansion and facilitates Treg cell differentiation and distal TCR signaling
Source: Commun Biol. 2022 Mar 4;5:206. doi: 10.1038/s42003-022-03129-x (PMC8897436; doi:10.1038/s42003-022-03129-x)
Supplement: Supplementary file 5 — Reporting Summary [file 42003_2022_3129_MOESM5_ESM.pdf]

nature portfolio

Corresponding author(s): Koko katagiri, Ph.D.

Last updated by author(s): January 31, 2022

Reporting Summary

Nature Portfolio wishes to improve the reproducibility of the work that we publish. This form provides structure for consistency and transparency in reporting. For further information on Nature Portfolio policies, see our Editorial Policies and the Editorial Policy Checklist.

Statistics

For all statistical analyses, confirm that the following items are present in the figure legend, table legend, main text, or Methods section.

n/a	Confirmed	
☐	☒	The exact sample size (*n*) for each experimental group/condition, given as a discrete number and unit of measurement
☐	☒	A statement on whether measurements were taken from distinct samples or whether the same sample was measured repeatedly
☐	☒	The statistical test(s) used AND whether they are one- or two-sided<br>*Only common tests should be described solely by name; describe more complex techniques in the Methods section.*
☒	☐	A description of all covariates tested
☒	☐	A description of any assumptions or corrections, such as tests of normality and adjustment for multiple comparisons
☐	☒	A full description of the statistical parameters including central tendency (e.g. means) or other basic estimates (e.g. regression coefficient) AND variation (e.g. standard deviation) or associated estimates of uncertainty (e.g. confidence intervals)
☐	☒	For null hypothesis testing, the test statistic (e.g. *F*, *t*, *r*) with confidence intervals, effect sizes, degrees of freedom and *P* value noted<br>*Give P values as exact values whenever suitable.*
☒	☐	For Bayesian analysis, information on the choice of priors and Markov chain Monte Carlo settings
☒	☐	For hierarchical and complex designs, identification of the appropriate level for tests and full reporting of outcomes
☒	☐	Estimates of effect sizes (e.g. Cohen's *d*, Pearson's *r*), indicating how they were calculated

Our web collection on statistics for biologists contains articles on many of the points above.

Software and code

Policy information about availability of computer code

Data collection	Confocal: Leica TCS SP8,l: Leica TCS SP8, Flow cytometer:Beckman Coulter CytoFLEX, Histological examination: Olympus BX53F, Western blot film and gel scan: ImageQant LAS4000mini
Data analysis	Image analysis: Leica X, cellSens Standard, Adobe Photoshop CS5.1, FlowCytometry analysis: Kaluza analysis version 2.1, Other data analysis: Microsoft office Excel 2016

For manuscripts utilizing custom algorithms or software that are central to the research but not yet described in published literature, software must be made available to editors and reviewers. We strongly encourage code deposition in a community repository (e.g. GitHub). See the Nature Portfolio guidelines for submitting code & software for further information.

Data

Policy information about availability of data

All manuscripts must include a data availability statement. This statement should provide the following information, where applicable:

- Accession codes, unique identifiers, or web links for publicly available datasets
- A description of any restrictions on data availability
- For clinical datasets or third party data, please ensure that the statement adheres to our policy

All data are available from the corresponding author upon reasonable request.

Field-specific reporting

Please select the one below that is the best fit for your research. If you are not sure, read the appropriate sections before making your selection.

- [x] Life sciences
- [ ] Behavioural & social sciences
- [ ] Ecological, evolutionary & environmental sciences

For a reference copy of the document with all sections, see nature.com/documents/nr-reporting-summary-flat.pdf

Life sciences study design

All studies must disclose on these points even when the disclosure is negative.

Sample size	Exact details of sample size, with technical and biological replicantes are indicated for each experimental time-point analyzed. Sampling sizes were determined based on previous studies, our experience and the number of knockout animals of appropriate age to reveal statistical significance (Ishihara et al., 2015). No statistical method was used to predetermine sample size.
Data exclusions	We excluded samples only for technical reasons, e.g. insufficient cell number upon isolation procedure. No obtained data were excluded.
Replication	All experiments were done at least three independent experiments. Specifics were indicated in the figure legends. All attempts at replications were successful.
Randomization	Animals with appropriate genotype were randomly assigned to each experimental groups.
Blinding	The scoring of the experiments was done in a blinded fashion.

Reporting for specific materials, systems and methods

We require information from authors about some types of materials, experimental systems and methods used in many studies. Here, indicate whether each material, system or method listed is relevant to your study. If you are not sure if a list item applies to your research, read the appropriate section before selecting a response.

Materials & experimental systems

n/a	Involved in the study	
☐	☒	Antibodies
☐	☒	Eukaryotic cell lines
☒	☐	Palaeontology and archaeology
☐	☒	Animals and other organisms
☒	☐	Human research participants
☒	☐	Clinical data
☒	☐	Dual use research of concern

Methods

n/a	Involved in the study	
☒	☐	ChIP-seq
☒	☐	Flow cytometry
☒	☐	MRI-based neuroimaging

Antibodies

Antibodies used

The following antibodies used for flow cytometry:
Purified anti-CD3 (Biolegend #100340, lot B302116); purified anti-CD28 (Biolegend #102116, lot B311420); fluorescein isothiocyanate (FITC)-conjugated anti-CD3 (Biolegend #100306, lot B241616), -CD19 (TONBO #35-0193-U100, lot C0193092117353), -B220 (Biolegend #103206, lot B314139), -NK1.1 (BD #553164, lot 80219), -CD62L (Biolegend #104405, lot B307924), -Helios (Biolegend #137204, lot B266829), -IFNg (Biolegend #505806, lot B266185); phycoerythrin (PE)- conjugated anti-CD8 (Biolegend #100708, lot B268831), -CD11c (Biolegend #117307, lot B212618), -CD44 (TONBO #50-0441-U100, lot C0441061218503), -CD64 (Biolegend #139315, lot B330234), -F4/80 (Biolegend #123107, lotB320936), -Foxp3 (Invitrogen/eBioscience #12-5773-80, lot 1984158), -RORgt (BD #562607, lot 5079716), -GATA3 (eBioscience #12-9966-41, lot E11591-1632), -IL-17F (Biolegend #517007, lot B280035); phycoerythrin -cyanin 7 (PE-Cy7)-conjugated anti-CD3 (TONBO #60-0031-U025, lot C0031092415602), -CD11b (Biolegend #101215, lot B324803), -CD44 (Biolegend #103030, lot B308091), -CTLA-4 (Biolegend #106313, lot B290849), -T-bet (eBioscience #25-5825-80, lot E15137-105), -IL-17A (Biolegend #506921, lot B308530); allophycocyanin-conjugated anti-CD4 (Biolegend #100516, lot B261590), -CD44 (Invitrogen #17-0441081, lot 2093724), -CD62L (Biolegend #104411, lot B282478), -MHC[illegible] (e-Bioscience #17-5321-81, lot E07293-1632), -Foxp3 (Invitrogen/eBioscience #17-5773-82); Brilliant VioletTM 421 (BV421)-conjugated anti-CD4 (Biolegend #100437, lot B310160), -CD45.1 (Biolegend #110731, lot B305752), -CD80 (Biolegend #104725, lot B308956), -CD86 (Biolegend #105031, lot B316212), -IL-10 (Biolegend #505021, lot B275076); Brilliant VioletTM 711 (BV711)-conjugated anti-CD3 (Biolegend #100349, lot B275433), -CD45.2 (Biolegend #109847, lot B305857), -CD103 (Biolegend #121435, lot B325971); Alexa Fluor 488 -Foxp3 (Biolegend #320011, lot B273261)
To detect live cell, the Zombie NIR™ Fixable Viability Kit (Biolegend #423105), Zombie Green™ Fixable Viability Kit (Biolegend #423111) were used.

The following antibodies used for western blotting :
anti-ZAP70 (Cell Signaling #3165T, lot 7), -pZAP70 (Cell Signaling #2701T, lot 10), -SLP76 (Cell Signaling #4958S, lot 2), -pSLP76 (Cell

Signalling # 14770S, lot 1), -PLCg (Cell Signaling # 5690T, lot 1), -pPLCg (Cell Signaling #2821T, lot 9), -c-Jun (Cell Signaling # 9165T, lot 9), -p-c-Jun (Cell Signaling # 3270T, lot 5), -Myc (Millipore # 50095, lot 2739392), -ERK (Cell Signaling #3165P, lot 6), -p-ERK (Cell Signaling # 4370S, lot 17), -HS1 (Cell Signaling #3892S, lot 1), -pHS1 (Cell Signaling # 8714S, lot 1); peroxidase-conjugated goat anti-mouse IgG (Cell Signaling #7076S, lot 33), peroxidase-conjugated goat anti-rabbit IgG (Cell Signaling #7074S, lot 28)

The following antibodies used for immunoflorescence:
anti-STIM-1 (Cell Signaling # 5668S, lot 3), -NFAT1(Cell Signaling #5861S, lot 4), pZAP70 (Cell Signaling #2701T, lot 10), -pPLCg(Abcam #ab76031), -WASP (Santa cruz #sc-13139, lot J1515), -SLP76 (pY128) PE (BD #558437, lot 9057736), -RAPH1 (invitrogen #PA5-110270, lot WH3347383), -RIAM (Abcam #EPR2806); Alexa Fluor 633–conjugated anti-rabbit IgG (Invitrogen #A21071, lot 2041628); and Alexa Fluor 633–conjugated anti-mouse IgG (Invitrogen #A21052, lot 1906490)

Validation

All primary antibodies used in this study were validated by the supplier/manufacture for use in flow cytometry, western blotting or immunofluorescence applications respectively.

Eukaryotic cell lines

Policy information about cell lines

Cell line source(s)	3A9 T cell hybridoma, CHO K1 cells
Authentication	3A9 T cell hybridoma was authenticated by Dr. P. M. Allen (J.Exp.Med., 1985). CHO K1 cells were from RIKEN BRC (cat#RCB0285, RRID: CVCL_0214; https://cellbank.brc.riken.jp/cell_bank/CellInfo/?cellNo=RCB0285&lang=Ja).
Mycoplasma contamination	All cell lines are free of mycoplasma contamination.
Commonly misidentified lines (See ICLAC register)	No cells in our study is listed in ICLAC.

Animals and other organisms

Policy information about studies involving animals; ARRIVE guidelines recommended for reporting animal research

Laboratory animals	Experiments using animals were performed on the following mice strains. C57BL/6, T-cell-specific Rap1a/b knockout mice, IL-17A knockout mice. Males and females age and sex matched between 2-14 weeks were used.
Wild animals	The study did not involve wild animals.
Field-collected samples	The study did not involve field-collected samples.
Ethics oversight	All animal experiments were conducted in accordance with the Regulations for the Care and Use of Laboratory Animals at Kitasato University, and the protocols used in the present study were ethically approved by the Institutional Animal Care and Use Committee at Kitasato University.

Note that full information on the approval of the study protocol must also be provided in the manuscript.
